# A proof-of-principle study for the point-of-care detection of ESBL (CTX-M) by NG-Test^®^ CTX-M MULTI lateral flow assay in urine samples using a simplified method for use in a resource-limited setting

**DOI:** 10.1093/jacamr/dlae103

**Published:** 2024-07-03

**Authors:** Dennis Nurjadi, Arnaud Chalin, Susanne Hauswaldt, Linus Olson, Mattias Larsson, Åse Östholm, Thirumalaisamy P Velavan, Sébastien Boutin, Jan Rupp, Lennart E Nilsson, Håkan Hanberger

**Affiliations:** Department of Infectious Diseases and Microbiology, University of Lübeck and University Hospital Schleswig-Holstein Campus Lübeck, Ratzeburger Allee 160, 23562 Lübeck, Germany; German Center for Infection Research (DZIF), Partner Site Hamburg-Lübeck-Borstel-Riems, Lübeck, Germany; Vietnamese German Center for Medical Research (VG-CARE), Hanoi, Vietnam; NG Biotech, R&D Department, Guipry, France; Department of Infectious Diseases and Microbiology, University of Lübeck and University Hospital Schleswig-Holstein Campus Lübeck, Ratzeburger Allee 160, 23562 Lübeck, Germany; Department of Women’s and Children’s Health, Karolinska Institutet, Stockholm, Sweden; Department of Global Public Health, Karolinska Institutet, Stockholm, Sweden; Training and Research Academic Collaboration (TRAC), Sweden, Vietnam; Department of Global Public Health, Karolinska Institutet, Stockholm, Sweden; Training and Research Academic Collaboration (TRAC), Sweden, Vietnam; Department of Infectious Diseases in Region Östergötland, Linköping, Sweden; Department of Biomedical and Clinical Sciences, Linköping University, Linköping, Sweden; Vietnamese German Center for Medical Research (VG-CARE), Hanoi, Vietnam; Institute of Tropical Medicine, Universitätsklinikum Tübingen, Tübingen, Germany; Faculty of Medicine, Duy Tan University, Da Nang, Vietnam; Department of Infectious Diseases and Microbiology, University of Lübeck and University Hospital Schleswig-Holstein Campus Lübeck, Ratzeburger Allee 160, 23562 Lübeck, Germany; German Center for Infection Research (DZIF), Partner Site Hamburg-Lübeck-Borstel-Riems, Lübeck, Germany; Airway Research Center North (ARCN), German Center for Lung Research (DZL), Lübeck, Germany; Department of Infectious Diseases and Microbiology, University of Lübeck and University Hospital Schleswig-Holstein Campus Lübeck, Ratzeburger Allee 160, 23562 Lübeck, Germany; German Center for Infection Research (DZIF), Partner Site Hamburg-Lübeck-Borstel-Riems, Lübeck, Germany; Department of Biomedical and Clinical Sciences, Linköping University, Linköping, Sweden; Training and Research Academic Collaboration (TRAC), Sweden, Vietnam; Department of Infectious Diseases in Region Östergötland, Linköping, Sweden; Department of Biomedical and Clinical Sciences, Linköping University, Linköping, Sweden

## Abstract

**Background:**

The rise of extended-spectrum β-lactamase-producing Enterobacterales (ESBL-E) in low- and middle-income countries limits treatment options, leading to the frequent use of broad-spectrum antibiotics. Reducing time-to-result for a urinary infection can facilitate correct antibiotic treatment and support antimicrobial and diagnostic stewardship measures. This study compared two simplified enrichment methods for detecting CTX-M directly from urine specimens.

**Methods:**

Two enrichment methods, namely centrifugation of 2 mL urine and filtration of 1 mL urine using the DirecTool adaptor, were compared using 20 culture-positive urine samples (20 suspected ESBL-E and 20 non-ESBL-E). CTX-M production was detected using a lateral flow assay (LFA), NG-Test^®^ CTX-MMULTI. The presence of *bla*_CTX-M_ genes was confirmed by whole-genome sequencing (WGS).

**Results:**

The results of both enrichment methods were identical, with a sensitivity of 87.5% and a specificity of 100%. In 19/20 (95%) of the urine samples, the results of the CTX-M LFA were identical with the phenotypic confirmation and WGS. Both methods could detect ESBL-E bacteriuria with ≥10^4^ cfu/mL. All ESBL-E-negative samples were identified accurately. Both enrichment methods yielded negative results in one ESBL-E-positive (CTX-M-15) sample despite phenotypic and genotypic confirmation of ESBL production. High leukocyte count (>500 cells/µL), the presence of boric acid or polymicrobial samples did not appear to impact the performance of both enrichment methods.

**Conclusions:**

Our study underscores the feasibility of directly detecting CTX-M in urine. Simplified enrichment methods, particularly with a filtration kit, enhance the assay’s practicality, rendering it suitable for use in primary care, emergency departments or remote laboratories without sophisticated equipment.

## Background

Antimicrobial resistance (AMR) is an ongoing global challenge, with the greatest impact in low- and middle-income countries (LMICs).^1,2^ Overuse of broad-spectrum antibiotics has been identified as a major catalyst for the emergence and spread of AMR in LMICs. Although efforts are being made to implement antimicrobial stewardship (AMS) measures to curb overuse and discourage unwarranted antibiotic prescribing, the effectiveness of AMS programmes is hampered by the lack of rapid and reliable microbiological diagnostics.^3,4^

Urinary tract infections (UTIs) are one of the most common causes of infection and account for a significant proportion of antibiotic use worldwide.^5,6^ With the emergence and spread of extended-spectrum β-lactamase-producing Enterobacterales (ESBL-E) in LMICs, the treatment options are limited, and physicians often resort to broad-spectrum antibiotics to anticipate the high local or regional prevalence of drug-resistant bacteria.^6,7^ The prompt initiation of correct antibiotic therapy is critical in febrile UTIs.^8^ In LMICs, as ESBL-producing *Escherichia coli* or *Klebsiella pneumoniae* are commonly encountered as uropathogens, physicians often rely on their experience or local epidemiology to select a treatment choice that is likely to be effective, leading to a tendency to overprescribe broad-spectrum antibiotics as a precaution.^9^

Expediting the time-to-result for urine diagnostics can significantly assist physicians in making informed decisions regarding antibiotic prescriptions.^3^ Point-of-care diagnostic tests (POCTs), specifically using a lateral flow assay (LFA) to identify CTX-M beta-lactamases, a predominant mechanism underlying the ESBL phenotype in *E. coli*, directly from urine specimens, have been proposed for this purpose.^10–12^ However, this approach may involve enriching or pre-incubating 10 mL urine samples, demanding sophisticated instrumentation and workflow, including more hands-on time to process the samples, thus hampering the POCT approach.^11^ In this study, our objective was to explore whether the procedure for detecting ESBL-E directly from urine specimens can be simplified for use in resource-limited settings. As a proof-of-principle, we conducted a head-to-head comparison of two enrichment methods, employing centrifugation and the DirecTool adapter filtration system.

## Methods

### Collection of clinical urine specimens

Rest clinical urine specimens were collected from the routine microbiology laboratory. Twenty of the urine samples with suspected ESBL-E, defined as resistance to penicillins, second- and third-generation cephalosporins, and 20 non-ESBL-E urine samples were chosen at random. The specimens were stored at 4°C–8°C after inoculation and collected for this study after 24–48 h. The detailed procedures of the routine microbiological diagnostic (culture, species identification, AST, determination of leukocyte count) as well as the sample size calculation for this proof-of-principle study are provided in the Supplementary Appendix (available as Supplementary data at *JAC-AMR* Online).

### Direct testing for CTX-M from urine specimens

**Figure 1.**
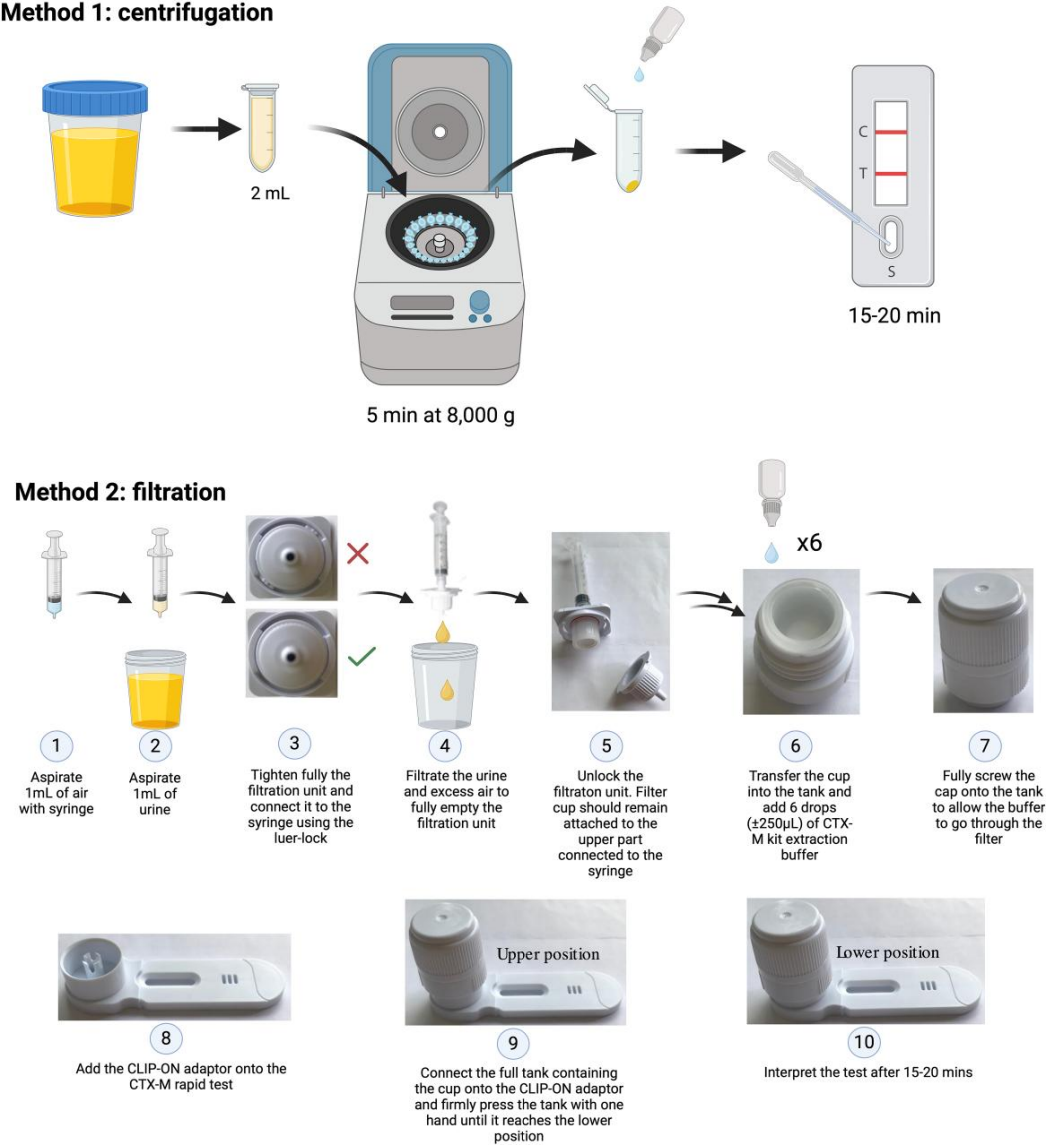
Schematic diagram of the urine processing methods to detect ESBL directly from urine specimen using NG-Test^®^ CTX-M MULTI LFA. For the centrifugation method, the urine sample in a 2 mL reaction tube was centrifuged using Eppendorf^®^ MiniSpin at 12 000 rpm (±8000 g). The filtration method was performed using the DirecTool CLIP-ON adaptor. Illustrations for method 2 were adapted from the protocol provided by NG Biotech. Created on Biorender^®^.

The aim of this study was to compare two different methods of preparation, by centrifugation and filtration, for the detection of CTX-M in urine samples (Figure 1). In the centrifugation approach, 2 mL of urine was centrifuged at 12 000 rpm (±8000 g, Eppendorf^®^ MiniSpin) for 5 min, and the resulting supernatant was discarded. Approximately 150 µL of NG-Test^®^ extraction buffer was added to the pellet, followed by vortexing to mix thoroughly. A 100 µL suspension of this mixture was immediately added to the sample well of the LFA cassette, and the results were interpreted after the control band became visible (±15–20 min). The filtration using NG-Test ^®^ DirecTool adapter was performed according to the manufacturer’s protocol with 1 mL urine (Figure 1).

### Whole-genome sequencing

DNA was extracted using DNeasy Blood and Tissue Kit (Qiagen GmbH, Germany), and library preparation (Illumina DNA prep kit) was performed following the manufacturer’s instructions. Sequencing was done on a MiSeq Illumina platform (short-read sequencing, 2 × 300 bp). Post-sequencing quality checks and assembly were performed as described previously.^13^ Draft genomes were checked for AMR genes using Abricate with NCBI, CARD, ARG-ANNOT, ResFinder and MEGARES databases.

### Statistical analysis

Descriptive statistics and performance of diagnostic tests (specificity and sensitivity) were analysed using Stata18 (StataCorp).

### Ethical clearance

The use of leftover samples for routine microbiological diagnostics did not require additional individual consent. The ethical committee of the University of Lübeck was consulted and waived individual consent (2022-620).

## Results

We randomly selected 40 culture-positive urine samples with suspected ESBL (*n* = 20) and non-ESBL Enterobacterales (*n* = 20) for this study. The molecular detection of *bla*_CTX-M_ by WGS was considered the true diagnosis for assessing the performance of both enrichment methods. One *E. coli* isolate (UR2) could not be recultured on the ESBL selective medium for WGS. Overall, the CTX-M MULTI LFA showed concordance with the microbiological culture in 38 out of 40 samples (95%). Of the 20 suspected ESBL producers, 17 were identified as *E. coli*, two as *K. pneumoniae* and one as *Citrobacter freundii*. WGS analysis showed that ST131 and ST88 were the predominant sequence types; six *E. coli* strains belonged to sequence type 131 (ST131) and two to ST88 (Table 1). Sixteen isolates harboured *bla*_CTX-M_ genes. The predominant ESBL gene detected was *bla*_CTX-M-15_ (11/16, 68.8%), followed by *bla*_CTX-M-1_ (3/16, 18.8%). Other CTX-M genes detected were *bla*_CTX-M-27_ and *bla*_CTX-M-65_ (1/16, 6.3% each). Other antibiotic resistance genes are summarized in Supplementary Table S1.

**Table 1.** Overview of urine specimen and rapid test performance to detect CTX-M

	NR	Sample type	Leukocyte count (per µL)	Polymicrobial	Enterobacterales^a^			Other species	NG-Test^®^ CTX-M MULTI			ESBL confirmation (genotypic and phenotypic)		
					species	quantification	ESBL phenotype^b^		centrifugation	filtration	direct from colony	growth on ESBL plate	WGS/CTX-M	MLST
**suspected ESBL**	UR1	urine	<10	yes	*E. coli*	10^6 cfu/ml	yes	*Enterococcus faecalis, Acinetobacter species*	pos	pos	pos	yes	CTX-M 15	ST131
	UR2	catheter urine	10–25	no	*E. coli*	10^4 cfu/ml	yes	—	pos	pos	n/a	n/a	n/a	n/a
	UR3	urine (bladder puncture)	<10	no	*E. coli*	10^4 cfu/ml	yes	—	pos	pos	pos	yes	CTX-M 27	ST131
	UR4	midstream urine	10–25	yes	*E. coli*	10^4 cfu/ml	yes	*E. faecalis*	pos	pos	pos	yes	CTX-M 15	ST131
	UR5	catheter urine	>500	yes	*E. coli*	10^6 cfu/ml	yes	*E. faecalis, Streptococcus anginosus*	pos	pos	pos	yes	CTX-M 15	ST131
	UR6	catheter urine	>500	no	*Citrobacter braakii*	10^4 cfu/ml	yes	—	neg	neg	neg	yes	no CTX-M detected, CMY 101	ST561
	UR7	midstream urine	>500	no	*E. coli*	10^6 cfu/ml	yes	—	pos	pos	pos	yes	CTX-M 1	ST88*
	UR8	midstream urine	10–25	no	*E. coli*	10^5 cfu/ml	yes	—	**neg^c^**	**neg**	**pos**	**yes**	CTX-M 15	ST131
	UR9	catheter urine	75	yes	*E. coli*	10^5 cfu/ml	yes	*Aerococcus urinae*	pos	pos	pos	yes	CTX-M 15	ST349
	UR10	midstream urine	>500	yes	*E. coli*	10^6 cfu/ml	yes	urogenital flora	pos	pos	pos	yes	CTX-M 1	ST88
	UR11	catheter urine	10–25	yes	*E. coli*	10^6 cfu/ml	yes	urogenital flora	pos	pos	pos	yes	CTX-M 15	ST131
	UR12	catheter urine	75	yes	*E. coli*	10^6 cfu/ml	yes	*Klebsiella pneumoniae, Candida krusei*	neg	neg	neg	yes	no CTX-M detected	ST131-1LV
	UR13	midstream urine	>500	no	*E. coli*	10^6 cfu/ml	yes		pos	pos	pos	yes	CTX-M 1	ST2015
	UR14	midstream urine	75	yes	*E. coli*	10^6 cfu/ml	yes	urogenital flora	neg	neg	neg	yes	no CTX-M detected	ST167
	UR15	catheter urine	>500	yes	*K. pneumoniae*	10^6 cfu/ml	yes	*Morganella morganii*	**neg^c^**	**neg**	**weak pos**	**yes**	CTX-M 15	ST432
	UR16	midstream urine	>500	yes	*E. coli*	10^6 cfu/ml	yes	urogenital flora	pos	pos	pos	yes	CTX-M 65	ST69
	UR17	midstream urine	<10	yes	*E. coli*	10^6 cfu/ml	yes	*Aerococcus urinae*	pos	pos	pos	yes	CTX-M 15	ST4891
	UR18	midstream urine	>500	yes	*K. pneumoniae*	10^5 cfu/ml	yes	*Staphylococcus epidermidis*	pos	pos	pos	yes	CTX-M 15	ST1662
	UR19	catheter urine	<10	no	*E. coli*	10^6 cfu/ml	yes		pos	pos	pos	yes	CTX-M 15	ST361
	UR20	midstream urine	75	no	*E. coli*	10^6 cfu/ml	yes		pos	pos	pos	yes	CTX-M 15	ST1193
**non-ESBL**	UR21	midstream urine	75	no	*E. coli*	10^4 cfu/ml	no	—	neg	neg	n/a	no	n/a	n/a
	UR22	midstream urine	10–25	no	*E. coli*	10^6 cfu/ml	no	—	neg	neg	n/a	no	n/a	n/a
	UR23	midstream urine	75	no	*E. coli*	10^6 cfu/ml	no	—	neg	neg	n/a	no	n/a	n/a
	UR24	midstream urine	10–25	no	*E. coli*	10^6 cfu/ml	no	—	neg	neg	n/a	no	n/a	n/a
	UR25	midstream urine	75	no	*E. coli*	10^6 cfu/ml	no	—	neg	neg	n/a	no	n/a	n/a
	UR26	midstream urine	75	no	*E. coli*	10^6 cfu/ml	no	—	neg	neg	n/a	no	n/a	n/a
	UR27	midstream urine	75	no	*E. coli*	10^6 cfu/ml	no	—	neg	neg	n/a	no	n/a	n/a
	UR28	midstream urine	>500	no	*E. coli*	10^6 cfu/ml	no	—	neg	neg	n/a	no	n/a	n/a
	UR29	midstream urine	>500	no	*E. coli*	10^5 cfu/ml	no	—	neg	neg	n/a	no	n/a	n/a
	UR30	catheter urine	>500	yes	*E. coli*	10^6 cfu/ml	no	*Enterococcus faecalis*	neg	neg	n/a	no	n/a	n/a
	UR31	catheter urine	75	no	*E. coli*	10^6 cfu/ml	no	—	neg	neg	n/a	no	n/a	n/a
	UR32	midstream urine	>500	no	*E. coli*	10^5 cfu/ml	no	—	neg	neg	n/a	no	n/a	n/a
	UR33	midstream urine	<10	yes	*urogenital flora*	10^2 cfu/ml	no	—	neg	neg	n/a	no	n/a	n/a
	UR34	catheter urine	>500	yes	*P. mirabilis*	10^5 cfu/ml	no	*Enterococcus faecalis*	neg	neg	n/a	no	n/a	n/a
	UR35	midstream urine	75	no	*E. coli*	10^5 cfu/ml	no	—	neg	neg	n/a	no	n/a	n/a
	UR36	midstream urine	<10	yes	*E. coli*	10^6 cfu/ml	no	*Enterobacter cloacae*	neg	neg	n/a	no	n/a	n/a
	UR37	catheter urine	10–25	yes	*K. pneumoniae*	10^6 cfu/ml	no	*Staphylococcus aureus*	neg	neg	n/a	no	n/a	n/a
	UR38	midstream urine	<10	no	*E. coli*	10^3 cfu/ml	no	urogenital flora	neg	neg	n/a	no	n/a	n/a
	UR39	catheter urine	75	no	*E. coli*	10^4 cfu/ml	no	none	neg	neg	n/a	no	n/a	n/a
	UR40	catheter urine	75	no	*E. coli*	10^6 cfu/ml	no	none	neg	neg	n/a	no	n/a	n/a

ESBL, extended-spectrum beta-lactamase; WGS, whole-genome sequencing; MLST, multi-locus sequence type; pos, positive; neg, negative; n/a, not applicable.

^a^Data acquired from the routine microbiological diagnostics.

^b^Isolates with suspected ESBL phenotype were defined as Enterobacterales with phenotypic resistance to penicillins and second- and third-generation cephalosporins, as determined by VITEK^®^2.

^c^Discrepant results between direct detection of CTX-M from urine samples and from bacterial colony are indicated in bold.

The NG-Test^®^ CTX-M MULTI results showed concordance between centrifugation and filtration pre-processing. Both pre-processing methods could detect 10^4^ cfu/mL CTX-M-producing *E. coli* compared to the lower detection limit of 10^5^ cfu/mL without pre-processing using spiked urine samples (Supplementary Methods and Figure S1). Three samples suspected of harbouring ESBL-producing *C. freundii* (sample UR6), *E. coli* (sample UR8) and *K. pneumoniae* (UR15) gave negative results in the LFA directly from urine samples. Further analysis of the resistome from the WGS data revealed that UR6 did not harbour any *bla*_CTX-M_ genes, justifying the negative LFA result. However, in the case of samples UR8 and UR15, the direct urine LFA yielded negative results, despite the presence of CTX-M-producing *E. coli* and positive LFA from culture (Table 1, Supplementary Figure S2). For UR15, however, the LFA from culture only produced a very weak positive band for CTX-M (Supplementary Figure S2).

Compared with microbial culture plus detection of *bla*_CTX-M_ by WGS as the gold standard, the direct urine testing, irrespective of pre-treatment methods, demonstrated a sensitivity of 87.5% (95% CI: 61.7%–98.4%) and a specificity of 100% (95% CI 83.9%–100%), a positive predictive value of 100% and a negative predictive value of 91.3%. Our proof-of-principle study suggests that high leukocyte count (>500 cells/µL), the presence of boric acid or polymicrobial samples did not have an impact on the performance of both enrichment methods (Table 1).

## Discussion

CTX-M detection directly from urine without enrichment has a limit of ≥10^5^ cfu/mL but exhibits weak band intensity, posing interpretation challenges for untrained staff (Supplementary Figure S1). Tang *et al*.^11^ recommend a prior enrichment step involving centrifugation of 10 mL urine at 3000 ×  g for 15 min, discarding the supernatant and additional incubation at 37°C for 20 min to enhance the sensitivity. However, this contradicts the POCT principle, as it requires equipment like a centrifuge and an incubator. This approach may be suitable for healthcare settings but proves challenging in primary care, outpatient clinics and decentralized settings in LMICs, where simple and affordable diagnostics are crucial. The versatility of this assay has been demonstrated by several studies that have evaluated the performance of this assay to detect CTX-M and carbapenemases from positive blood cultures, from urine specimens and even from rectal swabs with minimal modification of a short incubation period.^10,12,14^ In our study, we focused on direct testing from urine specimens as we believe this use case has the greatest potential to improve antibiotic prescribing in resource-limited settings.

In a study by Volland *et al*., the enrichment step consisted of filtering 3 mL of urine samples with a bacterial concentration of at least 10^5^ cfu/mL, using similar post-filtration processing as in our study. This approach demonstrated a sensitivity of 100% (95% CI: 91.19%–100%) and a specificity of 95% (95% CI: 95.6%–100%) for beta-lactamase detection.^11^ In our study, we observed a sensitivity of 87.5% with a specificity of 100%. A key difference in our research is that we used 1 mL of urine instead of 3 mL and included samples with a bacterial load ≥10^4^ cfu/mL. Furthermore, our study consisted of using a filtration device (DirecTool) adapted to an already available CE-marked product (NG-Test^®^ CTX-M MULTI), while the existing published data were obtained from a research-use-only device (BL DetecTool). Notably, our study demonstrated the ability to detect CTX-M in samples with a bacterial load of 10^4^ cfu/mL after enrichment. This finding is particularly relevant given that bacteriuria ≥10^4^ cfu/mL is generally considered a reliable threshold for UTI.^15^

Our study has certain limitations. The sample size for this comparative analysis was small (*n* = 20 per group), and the study was retrospective, with samples stored in the refrigerator at least overnight, which may affect the bacterial load and sensitivity of detection. This limitation is attributed to the low prevalence of ESBL-E causing UTI in our study setting. Despite these challenges, our results demonstrate that using a smaller volume (1 mL) provides reliable results. Further, our approach proved effective in detecting ESBL-producing bacteria at concentrations as low as 10^4^ cfu/mL. The strength of our study lies in the comprehensive comparison of different methods and incorporating CTX-M gene detection by WGS. Nevertheless, our data suggest that an easy-to-use microbiological POCT may be an alternative to the cumbersome conventional culture-based diagnostics. In this study, we only evaluated the feasibility of using the CTX-M MULTI panel, but there are other panels that could detect carbapenemase and *mcr* genes. Due to the low prevalence (<0.5%, based on unpublished routine data) of these genes in our study population, we were unable to validate the performance of these other panels.

### Conclusions

In conclusion, our study demonstrates the feasibility of direct detection of CTX-M in urine for rapid detection of ESBL-E, which is essential for the timely initiation of appropriate treatment, reducing precautionary use of broad-spectrum antibiotics and reducing selection pressure for antibiotic-resistant strains, particularly in resource-poor regions with a high burden of AMR. Simplified enrichment methods using a filtration kit increase the practicality of the assay, which can be used as a microbiological POCT on the bedside and is suitable for remote laboratories without electricity. Given the highly prevalent carbapenem resistance, it may be necessary to complement the CTX-M MULTI LFA with a rapid test for carbapenemase-producing Enterobacterales for implementation in LMICs. Larger clinical intervention studies are essential for further validation.

## Supplementary Material

dlae103_Supplementary_Data

## Data Availability

The sequencing data were uploaded to the NCBI GenBank under the bioproject number PRJNA1101205. Accession numbers are listed in the Supplementary Table.

## Supplementary data

Figures S1 and S2, Table S1 and Appendix are available as Supplementary data at *JAC-AMR* Online.
