# Supplementary material for: A proof-of-principle study for the point-of-care detection of ESBL (CTX-M) by NG-Test® CTX-M MULTI lateral flow assay in urine samples using a simplified method for use in a resource-limited setting
Source: JAC Antimicrob Resist. 2024 Jul 3;6(4):dlae103. doi: 10.1093/jacamr/dlae103 (PMC11220667; doi:10.1093/jacamr/dlae103)
Supplement: dlae103_Supplementary_Data [file dlae103_supplementary_data.zip › NG biotech supplementary data_R2.docx]

**Title:**

**A proof-of-principle study for the point-of-care detection of ESBL (CTX-M) by NG-Test® CTX-M MULTI lateral flow assay in urine samples using a simplified method for use in a resource-limited setting**

**Supplementary methods**

*Sample size calculation*

The study aims to test the hypothesis that the direct testing method is not inferior to the intended use of this test, i.e. detection from bacterial colonies. Therefore, parameters were set as follows; prevalence 50% (50% suspected ESBL and 50% non-ESBL as the sample selection criteria), expected sensitivity and specificity 0.99, precision of 0.05, confidence level 100 (1-α) of 95% and no dropout rate, the sample size required for sensitivity and specificity was calculated as n=31. The sample size calculation was performed using a previously published formula by Buderer (<https://wnarifin.github.io/ssc/sssnsp.html>).^1^

*Routine microbiological testing of urine samples*

Urine samples are collected in boric acid-containing urine monovettes (Sarstedt, Germany) in the routine microbiology laboratory. Using an automated streaking device (WASP®, Copan), 10 µL of urine were inoculated onto Columbia CNA agar and ChromID CPS agar (Biomérieux, Germany). Plates were incubated for at least 18 hours at 35±1°C before reading. Quantification was performed using a semi-quantification approach adapted to the streak pattern. Suspected uropathogens with significant colony counts (≥ 10³cfu/mL) were selected for identification and susceptibility testing using Vitek®2 (Biomérieux). Susceptibility testing results were interpreted according to EUCAST clinical breakpoints v13 (2023).

*Phenotypic testing for ESBL*

Leftover urine samples for routine microbiology were stored at 4°C until further use. Samples suspected of being ESBL-producing Enterobacterales, defined as phenotypically resistant to penicillins and beta-lactamase inhibitors (ampicillin/sulbactam or piperacillin/tazobactam) and third-generation cephalosporins, were recultured on ChromID ESBL Agar (Biomérieux) in addition to LFA CTX-M. Species identification of colonies growing on ESBL agar was performed by MALDI-TOF followed by whole genome sequencing.

*Determination of leukocyte count*

Upon receipt of the urine samples at the laboratory, a semi-quantitative leukocyte count was conducted using test strips (Combur² Test® LN, Roche). The test was visually interpreted following the manufacturer`s instructions with results categorised as <10/ µL, 10-25/ µL, approx. 75/ µL, >500/ µL or invalid.

*Bacterial preparation for urine spiking*

A clinical CTX-M-15-producing *Klebsiella pneumoniae* was grown overnight in LB medium under constant shaking at 150 rpm and 37°C. The liquid culture was centrifuged at 3000 rpm for 10 minutes to obtain a bacterial pellet. The supernatant was discarded, and the pellet was resuspended in sterile 0.9% NaCl for a washing step, followed by another centrifugation step and removal of the supernatant. The washed pellet was resuspended in 0.9% NaCl to reach a turbidity of 3.0 McFarland standard (≈1x10^9^ cfu/mL). CTX-M-negative fresh unfiltered urine was spiked using the bacterial suspension to reach the desired bacterial concentration (10^3^ to 10^6^ cfu/mL). For the direct testing using CTX-M Multi, 6 drops of CTX-M kit extraction buffer were added to 1 ml of spiked urine. Approximately 150 µL of this suspension was then transferred onto the LFA.

**Supplementary Figure S1. Direct detection of CTX-M using spiked urine samples. (a)** urine sample spiked with 10^6^ cfu/mL *Klebsiella pneumoniae* harbouring *bla*_CTX-M-15_ directly applied to the lateral flow cassette. As a positive control, CTX-M was detected from a bacterial colony of ESBL-producing *K. pneumoniae* cultivated on Columbia blood agar with 5% sheep’s blood. **(b)** Urine spiked with 10^3^  to 10^6^  cfu/mL CTX-M-15 producing *K. pneumoniae*. At concentrations of ≥10^5^ cfu/mL, a weak CTX-M band (T) was visible.

**
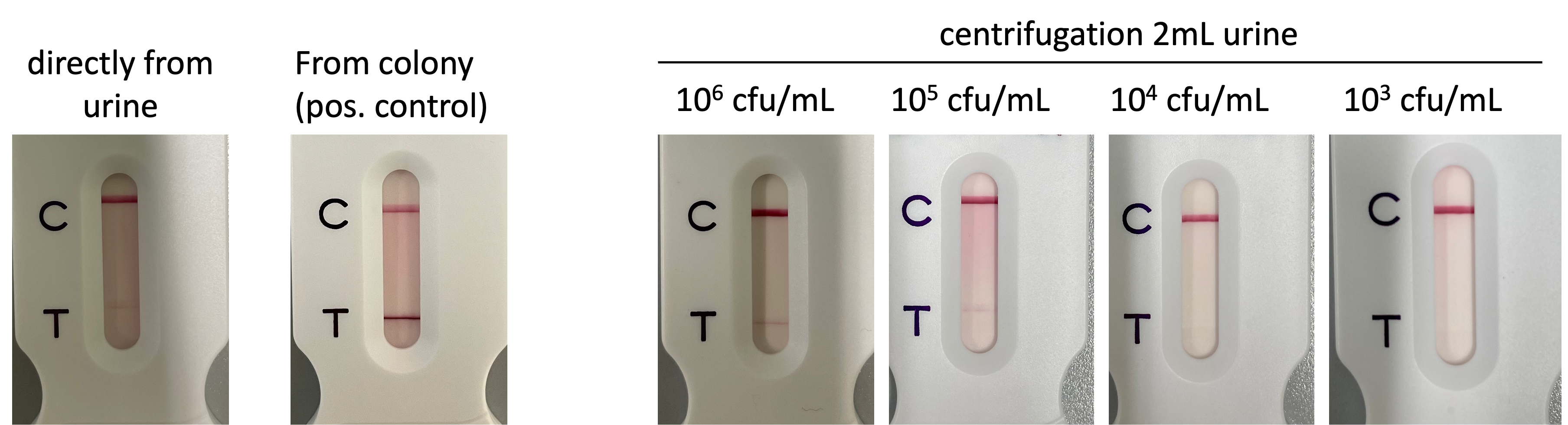
**

**Supplementary Figure S2. Comparison of LFA from centrifugation, filtration and direct colony in detecting CTX-M.** LFA from the bacterial colony was not available for *E. coli* UR2 since this strain was not able to be recultured on ESBL selective agar. Photo documentation for the direct colony LFA was missing for UR12 and UR14.


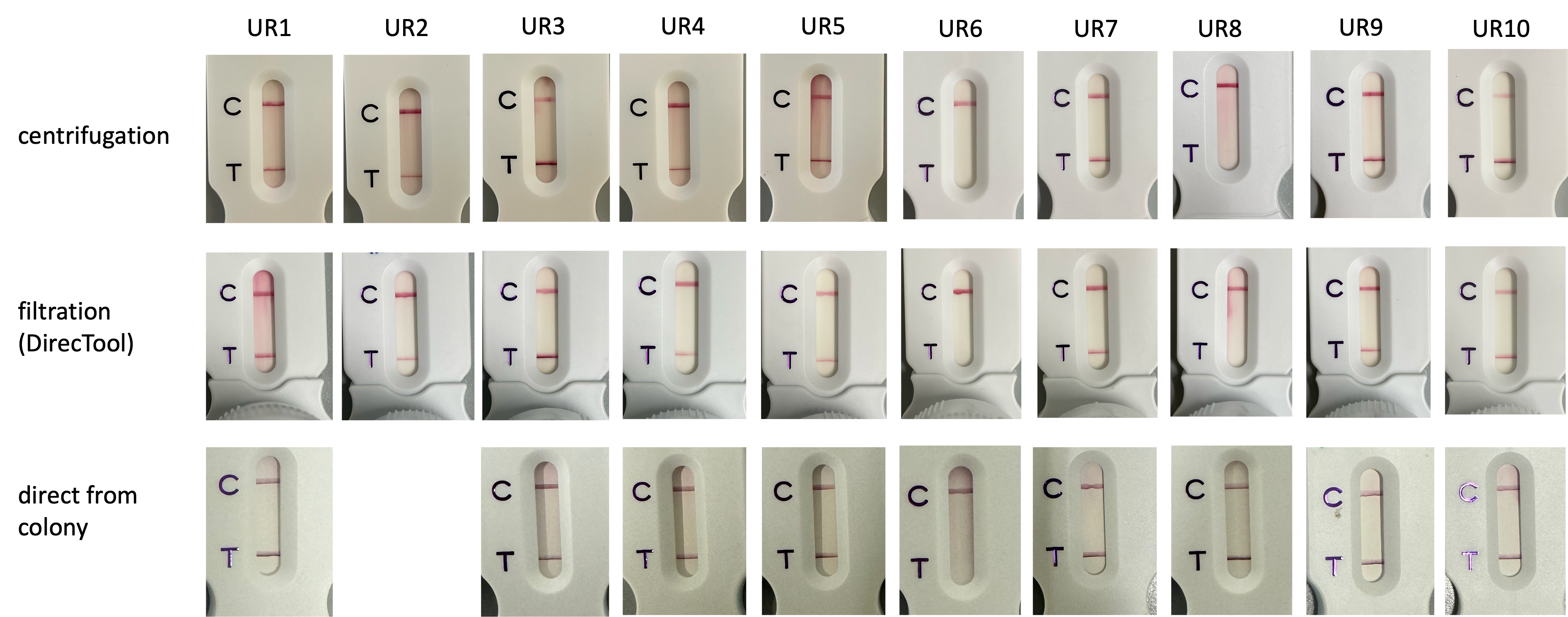


**
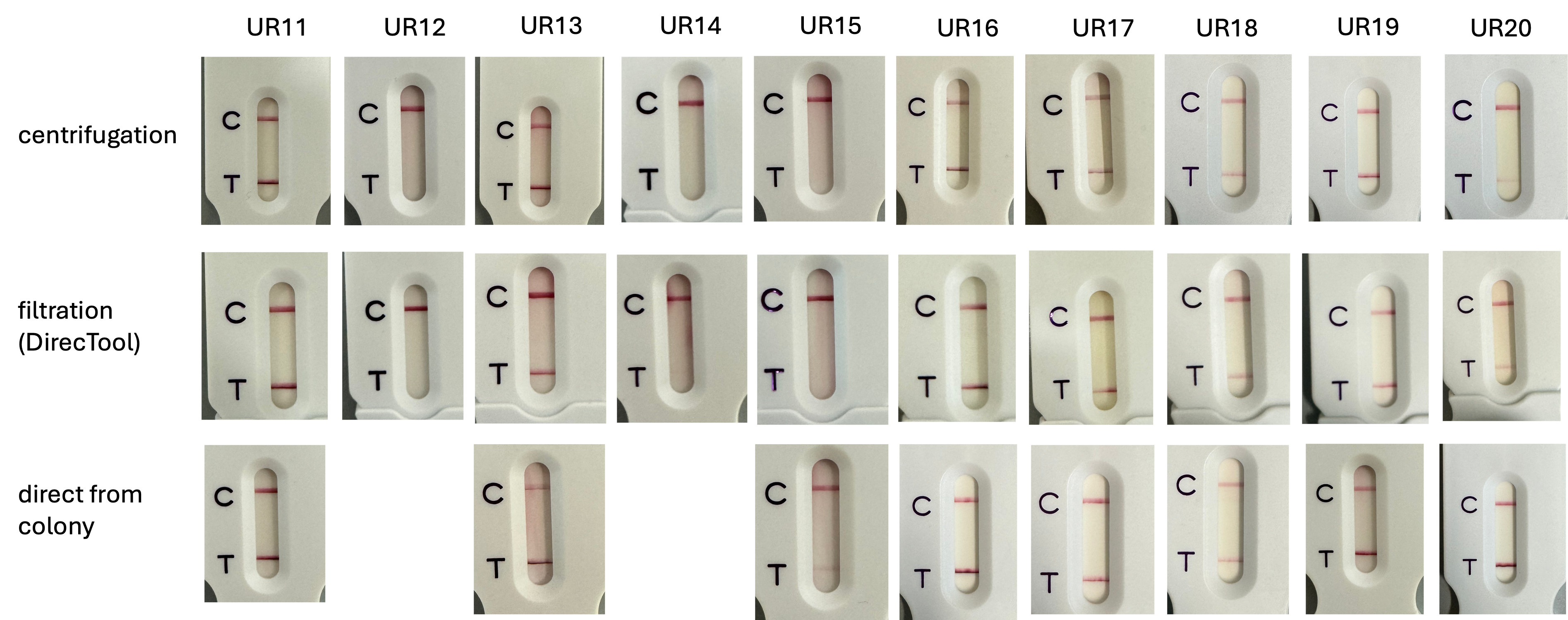
**

**Supplementary Table S1. Sequencing statistics and resistome of tested clinical Enterobacterales from urine specimen characterised using whole-genome sequencing.**

(see Excel file)

**References**

1. Buderer NM. Statistical methodology: I. Incorporating the prevalence of disease into the sample size calculation for sensitivity and specificity. *Acad Emerg Med* 1996; **3**: 895-900.
